# CENPB promotes the proliferation of hepatocellular carcinoma and is directly regulated by miR-29a

**DOI:** 10.18632/aging.205171

**Published:** 2023-11-02

**Authors:** Xuyang Wang, Laibang Luo, Youfu Zhang, Gang Liu, Zehong Fang, Zhidan Xu, Xuguang Hu

**Affiliations:** 1 Department of Organ Transplantation, Jiangxi Provincial People’s Hospital, The First Affiliated Hospital of Nanchang Medical College, Nanchang 330006, Jiangxi Province, China

**Keywords:** CENPB, hepatocellular carcinoma, tumor proliferation, prognostic biomarker, miR-29a

## Abstract

Hepatocellular carcinoma (HCC) is a significant global health concern as it ranks as the sixth most common malignant tumor and the third leading cause of cancer-related deaths. In this study, we analyzed the expression of centromere protein B (CENPB) mRNA in HCC using TCGA and GEO datasets. Immunohistochemistry (IHC) was performed to determine CENPB protein levels in 490 HCC patients. Our findings revealed higher expression of CENPB mRNA in HCC tissues across the three datasets. Additionally, as the pathological stage and histological grade advanced, CENPB expression increased. Patients with elevated levels of CENPB mRNA and protein demonstrated shorter overall survival (OS) and recurrence-free survival (OS). Notably, CENPB protein showed prognostic value in patients with stage I/II, AFP levels below 400 ng/ml, and tumor size less than 5 cm. Using multivariate regression analysis in 490 HCC patients, we developed nomograms to predict 1-year, 3-year, and 5-year OS and RFS. Knockdown of CENPB in Hep3B and MHCC97 cell lines resulted in significant inhibition of cell proliferation and invasion. Furthermore, bioinformatics analysis identified miR-29a as a potential negative regulator of CENPB expression, which was validated through a dual-luciferase reporter assay. In conclusion, our findings suggest that CENPB may serve as an oncogenic factor in HCC and is directly regulated by miR-29a, highlighting its potential as a promising therapeutic target.

## INTRODUCTION

Hepatocellular carcinoma (HCC) is a malignant tumor that originates from liver cells [1]. It is the most common type of liver cancer and is typically associated with factors such as chronic hepatitis, cirrhosis, and hepatitis B virus infection [2]. The incidence of HCC remains high worldwide and is a major cause of cancer-related deaths. It is well known that molecular markers play a significant role in prognostic evaluation of HCC [3]. These markers refer to molecular characteristics that undergo changes during the development and progression of HCC, and they can be used to predict patient prognosis and treatment response. In recent years, extensive research has been dedicated to identifying molecular markers associated with tumor prognosis, and this holds true for HCC as well. Several molecular markers have been identified for HCC, such as GPC3, SNRPD1, HCFC1 and PIVKA-II, among others [4–6]. However, their low specificity or sensitivity limits their widespread clinical utility. Alpha-fetoprotein remains the preferred molecular marker.

The CENPB gene encodes a protein known as Centromere Protein B, which plays a crucial role in the function of centromeres, the specialized regions of chromosomes essential for proper chromosome segregation during cell division [7]. CENPB plays a multifaceted role in tumorigenesis and tumor progression. It has been found to be upregulated or aberrantly expressed in various types of cancers, including breast cancer, lung cancer, and gastric cancer [8–10]. The dysregulation of CENPB in tumors suggests its potential involvement in promoting oncogenic processes. Cheng et al. found that CENPB is directly negatively regulated by miR-873-3p in lung squamous cell carcinoma, promoting the cell cycle process and facilitating tumor progression [11]. Zhang et al. discovered that the elevated expression level of CENPB affects the chemotherapy response and prognosis of breast cancer patients through upregulation of targets in the Pi3k/Akt/mTOR signaling pathway [12]. Furthermore, studies have indicated that LINC01123 acts as a sponge for miR-151a, thereby upregulating CENPB expression to enhance the radioresistance of glioma cells both *in vitro* and *in vivo* [13]. What is worth noting is that current research has found that antibodies targeting CENPB can prolong the survival of breast cancer patients [14]. Therefore, CENPB has shown promising clinical prognostic value in the development of various tumors and may be a potential therapeutic target. However, its role in HCC needs further research.

In this study, we initially identified higher expression of CENPB mRNA in HCC than adjacent normal liver tissues across three databases. Further analysis in a cohort of 490 HCC patients confirmed increased protein expression of CENPB, which was associated with an adverse prognosis. Interestingly, both the high expression of CENPB mRNA and protein have been identified as independent risk factors associated with poorer prognosis. Subsequently, we developed two nomogram models capable of quantitatively predicting overall survival (OS) and recurrence-free survival (RFS). *In vitro* experiments demonstrated that silencing CENPB expression significantly inhibited the migration, invasion, and proliferation capabilities of HCC cells. Furthermore, through bioinformatics analysis and dual-luciferase reporter assays, miR-29a was identified as an upstream regulator of CENPB expression.

## RESULTS

### CENPB mRNA is upregulated in HCC and indicates a poor prognosis

**Figure 1:**
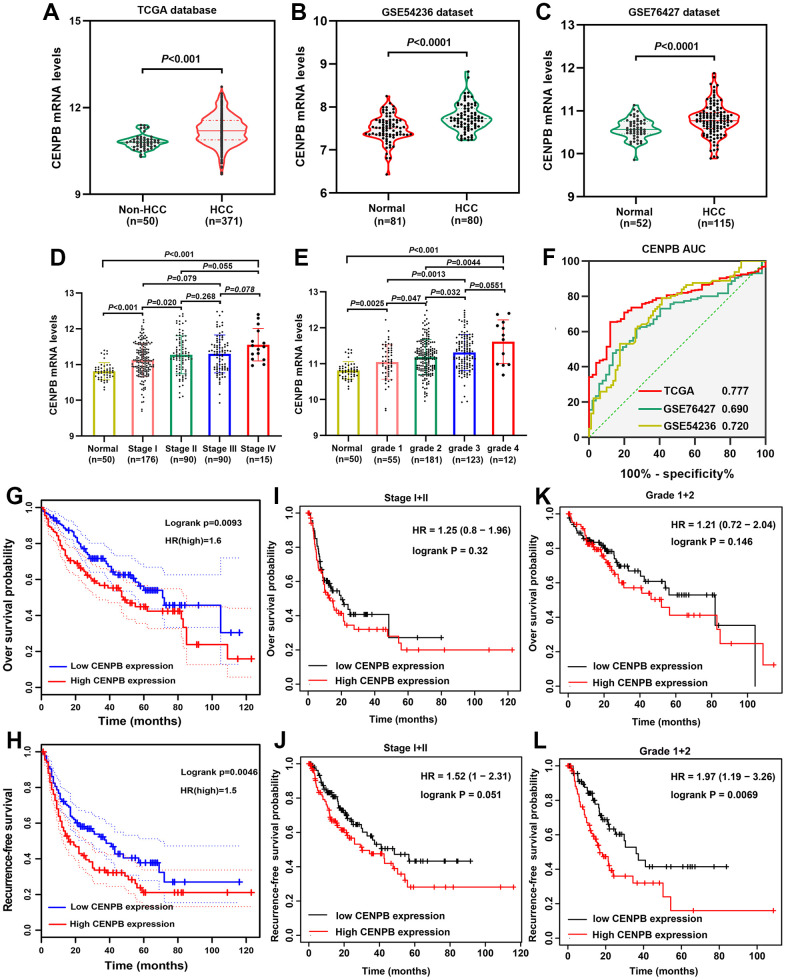
**Prognostic significance of CENPB mRNA in HCC.** (**A**–**C**) CENPB mRNA was found to be higher in HCC compared to normal liver tissues, as observed in the TCGA (**A**), GSE54236 (**B**), and GSE76427 (**C**) datasets. (**D**) The diagnostic potential of CENPB was assessed using ROC curves in the TCGA, GSE54236, and GSE76427 datasets. (**E**, **F**) CENPB mRNA levels exhibited a gradual increase with advancing tumor stage (**E**) and grade (**F**). (**G**, **H**) Elevated CENPB mRNA levels were associated with poor OS (**G**) and RFS (**H**). (**I**, **J**) High CENPB mRNA levels were predictive of poor OS (**I**) and RFS (**J**) specifically in stage I/II patients. (**K**, **L**) High CENPB mRNA levels predicted unfavorable OS (**K**) and RFS (**L**) in grade I/II patients.

We examined the CENPB mRNA in both HCC and adjacent normal liver tissues using TCGA, GSE54236, and GSE76427 datasets. Consistently, our findings revealed that CENPB mRNA was elevated in HCC tissues compared to adjacent normal liver tissues (Figure 1A–1C). Notably, in the TCGA dataset, CENPB mRNA levels exhibited a gradual increase in correlation with tumor stage and pathological grade (Figure 1D, 1E). Furthermore, ROC curve analysis indicated that CENPB mRNA exhibited favorable diagnostic efficiency across all three datasets, as reflected by the AUC values of 0.777, 0.692, and 0.720, respectively (Figure 1F). Survival analysis conducted in the GEPIA database indicated that elevated levels of CENPB mRNA were associated with unfavorable OS and RFS outcomes among HCC patients (Figure 1G, 1H). Intriguingly, the Kaplan-Meier plotter database’s survival curves demonstrated that even in patients with earlier tumor stages (Stage I+II) and lower pathological grades (Grade 1+2), increased expression of CENPB still correlated with poorer OS and RFS (Figure 1I–1L). Following that, we conducted an investigation into the connection between CENPB expression and clinical pathological characteristics within the TCGA database. Our findings demonstrated a significant correlation between elevated levels of CENPB mRNA and T stage (*P*=0.003), pathological staging (*P*=0.026), histologic grade (*P*=0.049), AFP (*P*=0.006), Child-Pugh grade (*P*=0.016), and vascular invasion (*P*=0.019) (Table 1). Moreover, a univariate Cox regression analysis identified pathologic stage, tumor status, vascular invasion, and CENPB mRNA as influential risk factors impacting both OS and RFS. Subsequently, a multivariate Cox regression analysis confirmed that tumor status, vascular invasion, and CENPB mRNA all exerted independent effects on OS and RFS. Furthermore, pathologic stage emerged as an independent risk factor specifically for RFS (Table 2).

**Table 1:** Correlation between CENPB mRNA and clinicopathologic features in 374 patients with hepatocellular carcinoma.

**Characteristics**	**CENPB mRNA expression**		**P-value**
	**Low (n=187)**	**High (n=187)**	
Age, median (IQR)	61 (52, 68)	61 (51, 69)	0.662
Gender, n (%)			1.000
Female	61 (16.3%)	60 (16%)	
Male	126 (33.7%)	127 (34%)	
T stage, n (%)			0.003
T1	107 (28.8%)	76 (20.5%)	
T2	37 (10%)	58 (15.6%)	
T3	38 (10.2%)	42 (11.3%)	
T4	3 (0.8%)	10 (2.7%)	
N stage, n (%)			1.000
N0	127 (49.2%)	127 (49.2%)	
N1	2 (0.8%)	2 (0.8%)	
M stage, n (%)			0.623
M0	131 (48.2%)	137 (50.4%)	
M1	1 (0.4%)	3 (1.1%)	
Pathologic stage, n (%)			0.026
Stage I	102 (29.1%)	71 (20.3%)	
Stage II	36 (10.3%)	51 (14.6%)	
Stage III	39 (11.1%)	46 (13.1%)	
Stage IV	2 (0.6%)	3 (0.9%)	
Tumor status, n (%)			0.129
Tumor free	110 (31%)	92 (25.9%)	
With tumor	70 (19.7%)	83 (23.4%)	
Age, n (%)			1.000
<=60	89 (23.9%)	88 (23.6%)	
>60	98 (26.3%)	98 (26.3%)	
Histologic grade, n (%)			0.049
G1	34 (9.2%)	21 (5.7%)	
G2	94 (25.5%)	84 (22.8%)	
G3	51 (13.8%)	73 (19.8%)	
G4	5 (1.4%)	7 (1.9%)	
Adjacent hepatic tissue inflammation, n (%)			0.490
None	68 (28.7%)	50 (21.1%)	
Mild	51 (21.5%)	50 (21.1%)	
Severe	11 (4.6%)	7 (3%)	
AFP (ng/ml), n (%)			0.006
<=400	120 (42.9%)	95 (33.9%)	
>400	23 (8.2%)	42 (15%)	
Child-Pugh grade, n (%)			0.016
A	108 (44.8%)	111 (46.1%)	
B	16 (6.6%)	5 (2.1%)	
C	1 (0.4%)	0 (0%)	
Vascular invasion, n (%)			0.019
No	119 (37.4%)	89 (28%)	
Yes	47 (14.8%)	63 (19.8%)	
Fibrosis score, n (%)			0.401
0	40 (18.6%)	35 (16.3%)	
1/2	12 (5.6%)	19 (8.8%)	
3/4	13 (6%)	15 (7%)	
5/6	45 (20.9%)	36 (16.7%)	

**Table 2:** Univariate and multivariate Cox regression analysis of overall survival and recurrence-free survival in 374 patients with hepatocellular carcinoma.

**Characteristics**	**OS**		**RFS**	
	**HR (95% CI)**	*P-value	**HR (95% CI)**	*P-value
Univariate analysis				
Age				
<=60 vs >60	0.960 (0.718-1.284)	0.783	1.205 (0.850-1.708)	0.295
Gender				
Female vs Male	0.982 (0.721-1.338)	0.909	0.793 (0.557-1.130)	0.200
Pathologic stage				
Stage I&II vs Stage III&IV	2.201 (1.591-3.046)	<0.001	2.504 (1.727-3.631)	<0.001
Tumor status				
With tumor vs Free tumor	11.342 (7.567-17.000)	<0.001	2.317 (1.590-3.376)	<0.001
Histologic grade				
G1&G2 vs G4&G3	1.152 (0.853-1.557)	0.355	1.091 (0.761-1.564)	0.636
Adjacent hepatic tissue inflammation				
None vs Mild&Severe	1.238 (0.867-1.768)	0.241	1.194 (0.734-1.942)	0.475
AFP (ng/ml)				
<=400 vs >400	1.045 (0.698-1.563)	0.832	1.075 (0.658-1.759)	0.772
Child-Pugh grade				
A vs C&B	1.395 (0.765-2.545)	0.277	1.643 (0.811-3.330)	0.168
Vascular invasion				
Yes vs No	1.676 (1.196-2.348)	0.003	1.344 (1.012-2.035)	0.013
CENPB mRNA				
Low vs High	1.544 (1.153-2.068)	0.004	1.475 (1.041-2.089)	0.029
Multivariate analysis				
Pathologic stage				
Stage I&II vs Stage III&IV	1.330 (0.877-2.018)	0.180	2.134 (1.437-3.170)	<0.001
Tumor status				
With tumor vs Free tumor	11.821 (7.546-18.519)	<0.001	1.863 (1.248-2.782)	0.002
Vascular invasion				
Yes vs No	1.546 (1.065-2.245)	0.022	1.274 (1.221-1.973)	0.033
CENPB mRNA				
Low vs High	1.286 (1.103-1.832)	0.033	1.351 (1.207-1.977)	0.037

Abbreviations: HR, hazard ratio; CI, confidential interval; AFP, alpha fetoprotein; OS, overall survival; RFS, recurrence-free survival. *P-Value<0.05 were considered statistically significant.

### High CENPB protein expression in HCC tissue predicts a poor prognosis

**Figure 2:**
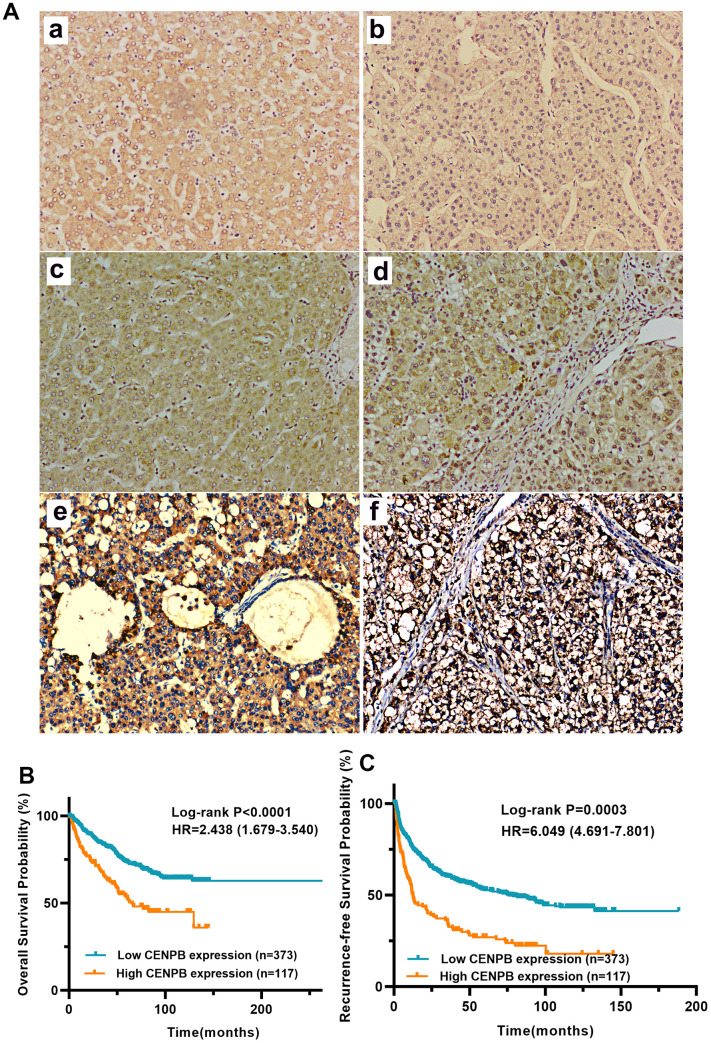
(**A**) CENPB protein expression and its prognostic value in a cohort with 490 HCC patients. (**A**) Representative IHC images of normal liver tissue (**a**) and HCC tissue with IHC scores 0 (**b**), 1 (**c**), 2 (**d**), 3 (**e**), and 4 (**f**), respectively, are shown below. (**B**, **C**) High expression of CENPB protein was found to be indicative of unfavorable OS (**B**) and RFS (**C**).

We utilized IHC staining to assess CENPB protein expression in 490 pairs of HCC and paracancer tissue samples. The findings exhibited predominant cytoplasmic localization of CENPB protein, with minimal presence in the cell nucleus. In alignment with the results obtained from bioinformatics analysis, CENPB protein demonstrated significantly elevated expression within HCC tissue compared to adjacent normal liver tissue (Figure 2A, a). Representative IHC staining images showcasing patients with varying scores were presented (Figure 2A, b-f). Subsequently, we examined the relationship between CENPB protein expression and clinical pathological data in the 490 HCC patients. The outcomes illustrated a positive correlation between high CENPB protein expression and age (*P*=0.036), tumor size (*P*<0.001), TNM staging (*P*=0.014), serum AFP levels (*P*=0.015), multiple nodules (*P*=0.015), vascular invasion (*P*=0.037), tumor encapsulation (*P*<0.001), tumor recurrence (*P*<0.001), and survival status (*P*<0.001) (Table 3). Furthermore, univariate Cox regression analysis identified tumor size, TNM stage, serum AFP levels, liver cirrhosis, and high CENPB protein expression as risk factors influencing OS and RFS. Additionally, the multivariate Cox regression analysis confirmed that serum AFP levels (*P*=0.049), liver cirrhosis (*P*<0.001), and high CENPB protein expression (*P*=0.014) were independent predictors of OS. As for RFS, liver cirrhosis (*P*<0.001) and high CENPB protein expression (*P*=0.002) were identified as independent predictive factors (Table 4). According to the IHC scoring, 117 patients exhibited high expression of CENPB protein, while 373 patients showed low expression. Subsequently, we performed survival analysis to investigate the correlation between protein expression and prognosis. The findings indicated that patients with elevated CENPB protein expression experienced reduced OS and RFS durations (Figure 2B, 2C).

**Table 3:** Correlation between CENPB protein expression and clinicopathologic features in 490 patients with hepatocellular carcinoma.

**Characteristics**		**N**	**CENPB level**		**χ^2^**	*P-Value
			**High(n)**	**Low(n)**		
Age (year)	>50	288	59	229	4.421	0.036
	<=50	202	58	144		
Gender	Male	424	104	320	0.733	0.392
	Female	66	13	53		
Tumor size (cm)	>5cm	213	78	135	33.657	0.000
	<=5cm	277	39	238		
TNM stage	I/II	34	14	20	6.015	0.014
	III	456	103	353		
Serum AFP level	>400ng/ml	164	50	114	5.926	0.015
	<=400ng/ml	326	67	259		
Liver cirrhosis	Yes	244	53	191	1.243	0.265
	No	246	64	182		
Multiple nodules	Yes	85	29	56	5.933	0.015
	No	405	88	317		
Tumor differentiation	Poor	41	8	33	0.469	0.493
	Moderate/well	449	109	340		
HBsAg	Positive	438	101	337	1.520	0.218
	Negative	52	16	36		
Vascular invasion	Yes	229	65	166	4.365	0.037
	No	261	52	207		
Tumor encapsulation	Yes	219	31	188	20.592	0.000
	No	271	86	185		
Recurrence	Yes	278	86	192	17.609	0.000
	No	212	31	181		
Survival status	Alive	178	59	119	13.212	0.000
	Dead	212	58	254		

**Table 4:** Univariate and multivariate Cox regression analysis of overall survival and recurrence-free survival in 490 patients with hepatocellular carcinoma.

**Characteristics**		**OS**	*P-Value	**RFS**	*P-Value
		**HR (95%CI)**		**HR (95%CI)**	
Univariate analysis					
Age (year)	>50 vs. <=50	0.875(0.650-1.178)	0.359	0.972(0.766-1.234)	0.816
Gender	Male vs. female	1.061(0.679-1.658)	0.795	1.267(0.877-1.832)	0.207
Tumor size (cm)	>5 vs. <=5	1.939(1.442-2.607)	<0.001	1.764(1.393-2.233)	<0.001
TNM stage	I/II vs. III	1.835(1.453-2.318)	<0.001	1.799(1.487-2.177)	<0.001
Serum AFP level	>400 vs <=400	1.632(1.210-2.200)	0.001	1.332(1.043-1.701)	0.022
Liver cirrhosis	Yes vs. no	1.388(1.194-1.614)	<0.001	1.308(1.107-1.544)	0.002
Multiple nodules	Yes vs. no	1.700(1.206-2.396)	0.002	1.593(1.194-2.124)	0.002
Tumor differentiation	Well vs. Moderate/Poor	0.973(0.564-1.681)	0.923	0.994(0.654-1.511)	0.977
HBsAg	Positive vs. negative	1.354(0.798-2.257)	0.262	1.154(0.776-1.716)	0.481
Vascular invasion	Yes vs. no	0.936(0.697-1.258)	0.663	1.020(0.806-1.291)	0.870
Tumor encapsulation	Yes vs. no	0.773(0.573-1.043)	0.092	0.776(0.611-0.985)	0.037
CENPB protein level	High vs. low	2.080(1.520-2.846)	<0.001	2.133(1.651-2.757)	<0.001
Multivariate analysis					
Tumor size (cm)	>5 vs. <=5	1.652(0.984-2.774)	0.058	1.294(0.859-1.949)	0.217
TNM stage	I/II vs. III	1.062(0.603-1.870)	0.835	1.329(0.849-2.081)	0.213
Serum AFP level	>400 vs <=400	1.367(1.001-1.867)	0.049	1.122(0.869-1.449)	0.376
Liver cirrhosis	Yes vs. no	1.356(1.175-1.563)	<0.001	1.364(1.178-1.579)	<0.001
Multiple nodules	Yes vs. no	1.235(0.707-2.159)	0.459	1.027(0.655-1.608)	0.909
Tumor encapsulation	Yes vs. no	0.843(0.617-1.150)	0.281	0.831(0.651-1.061)	0.137
CENPB protein level	High vs. low	1.588(1.100-2.293)	0.014	1.612(1.195-2.174)	0.002

Abbreviations: HR, hazard ratio; CI, confidential interval; AFP, alpha fetoprotein; TNM, tumor, node, metastasis; OS, overall survival; RFS, recurrence-free survival. *P-Value<0.05 were considered statistically significant.

### Elevated CENPB protein remains prognostically valuable in patients with early-stage, smaller size, and median/well differentiation

**Figure 3:**
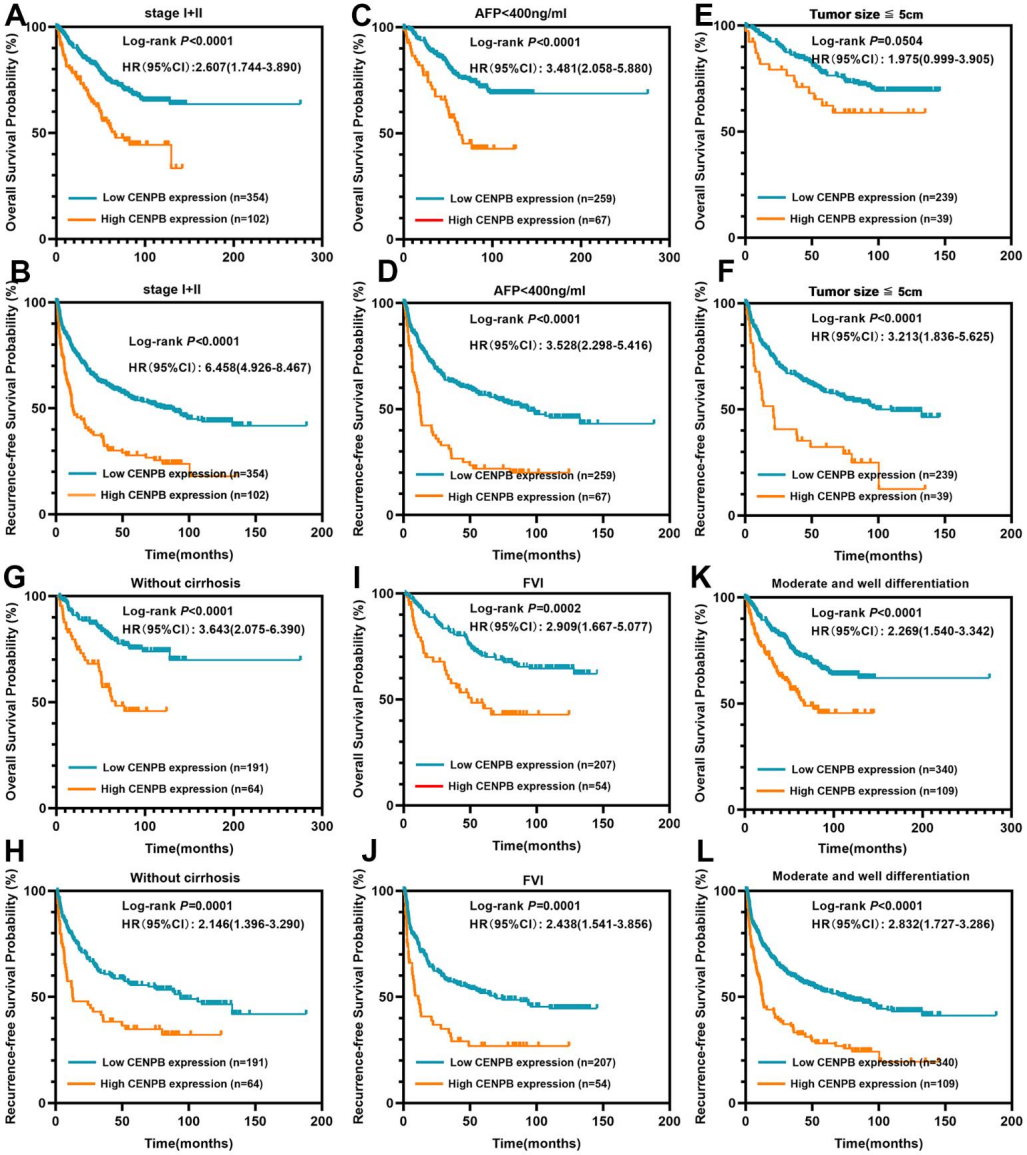
**CENPB protein levels were found to be associated with the prognosis of patients within early-stage subgroups.** (**A**, **B**) High CENPB protein expression predicted a shorter OS (**A**) and RFS (**B**) time for patients in stage I/II. (**C**, **D**) High CENPB protein expression predicted a shorter OS (**C**) and RFS (**D**) time for patients with AFP <400ng/ml. (**E**, **F**) High CENPB protein expression predicted a shorter OS (**E**) and RFS (**F**) time for patients with tumor size smaller than 5cm. (**G**, **H**) High CENPB protein expression predicted a shorter OS (**G**) and RFS (**H**) time for without cirrhosis patients. (**I**, **J**) High CENPB protein expression predicted a shorter OS (**I**) and RFS (**J**) time for patients with free of vascular invasion. (**K**, **L**) High CENPB protein expression predicted a shorter OS (**K**) and RFS (**L**) time for patients with median/well differentiation.

We proceeded to analyze the clinical prognostic significance of CENPB protein expression in patients characterized by low tumor staging, small tumor size, moderate/well differentiation and others. The findings demonstrated that elevated expression of CENPB protein was linked to inferior OS and RFS in patients exhibiting low tumor staging (Stage I+II, Figure 3A, 3B), AFP levels below 400ng/ml (Figure 3C, 3D), tumor diameter less than 5cm (Figure 3E, 3F), without of liver cirrhosis (Figure 3G, 3H), free of vascular invasion (FVI, Figure 3I, 3J), and moderate/well differentiation (Figure 3K, 3L). These results indicate that CENPB protein expression also holds favorable clinical prognostic value for early-stage tumors.

### Construction and validation of predictive nomogram

**Figure 4:**
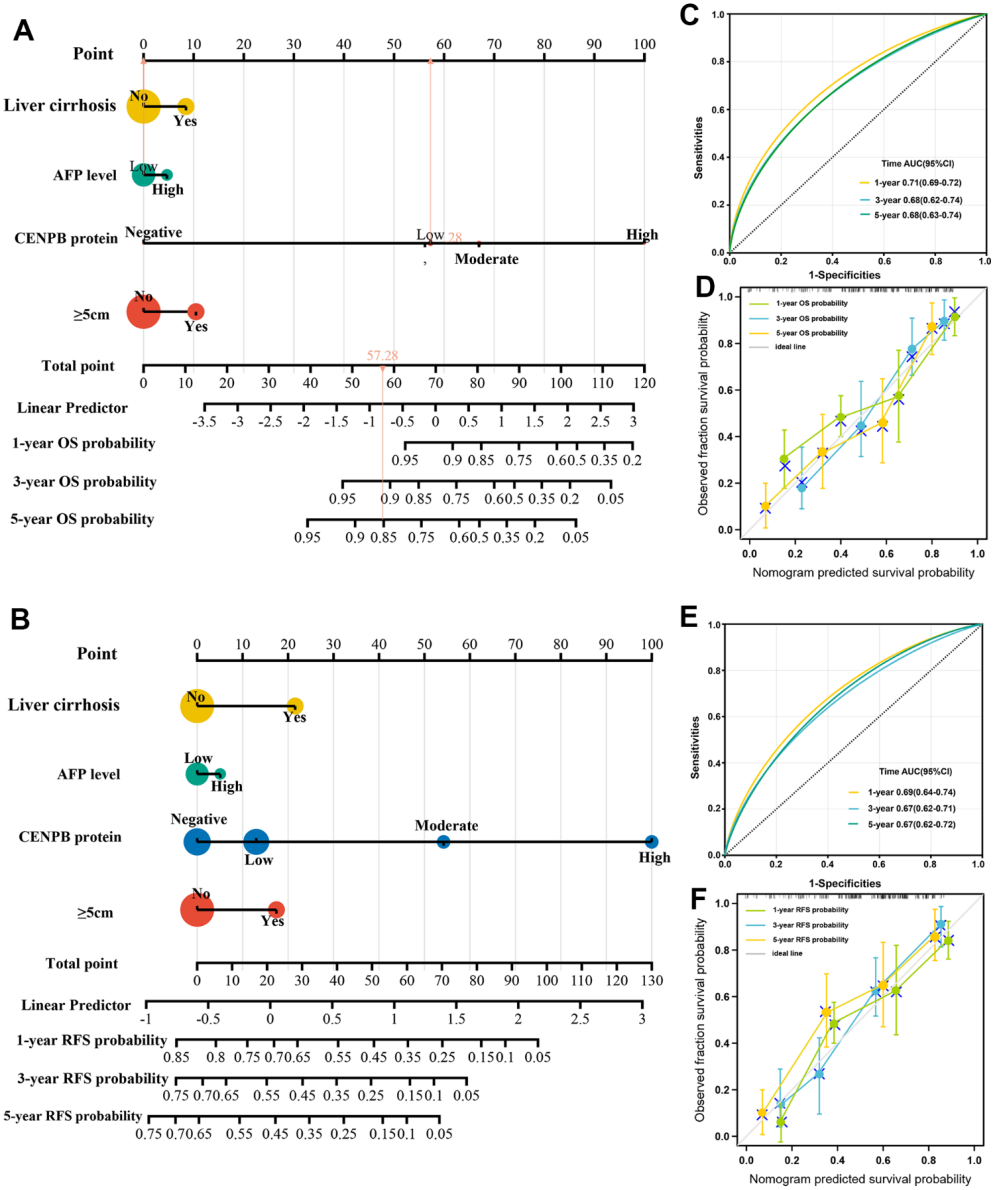
**Construction and validation of predictive nomogram.** (**A**, **B**) The nomograms for OS (**A**) and RFS (**B**) prediction incorporated independent risk factors that were identified through multivariate Cox regression analysis. (**C**, **D**) The AUC (**C**) and calibration (**D**) curves confirmed the precise predictive performance of the OS predicting nomogram. (**E**, **F**) The AUC (**E**) and calibration (**F**) curves confirmed the precise predictive performance of the RFS predicting nomogram.

In order to precisely predict the OS and RFS, we developed nomograms using the clinicopathologic parameter of the cohort with 490 HCC patients. The predictive accuracy was assessed through the time-dependent calibration curves and ROC curves. The nomograms for OS and RFS prediction incorporated independent risk factors that were identified through multivariate Cox regression analysis, including tumor diameter, liver cirrhosis, serum AFP levels, and CENPB protein expression (Figure 4A, 4B). The AUC values for 1-year, 3-year, and 5-year OS predictions were 0.71, 0.68, and 0.68, respectively (Figure 4C). The calibration curves confirmed the precise predictive performance of the OS predicting nomogram (Figure 4D). Likewise, the AUCs for predicting RFS at 1-year, 3-year, and 5-year were calculated as 0.69, 0.67, and 0.67, respectively (Figure 4E). The calibration curves demonstrated a close alignment between the observed and ideal curves, indicating the outstanding predictive performance of the RFS predicting nomogram (Figure 4F).

### CENPB inhibition suppresses proliferative and invasive capacities of HCC cells *in vitro*

**Figure 5:**
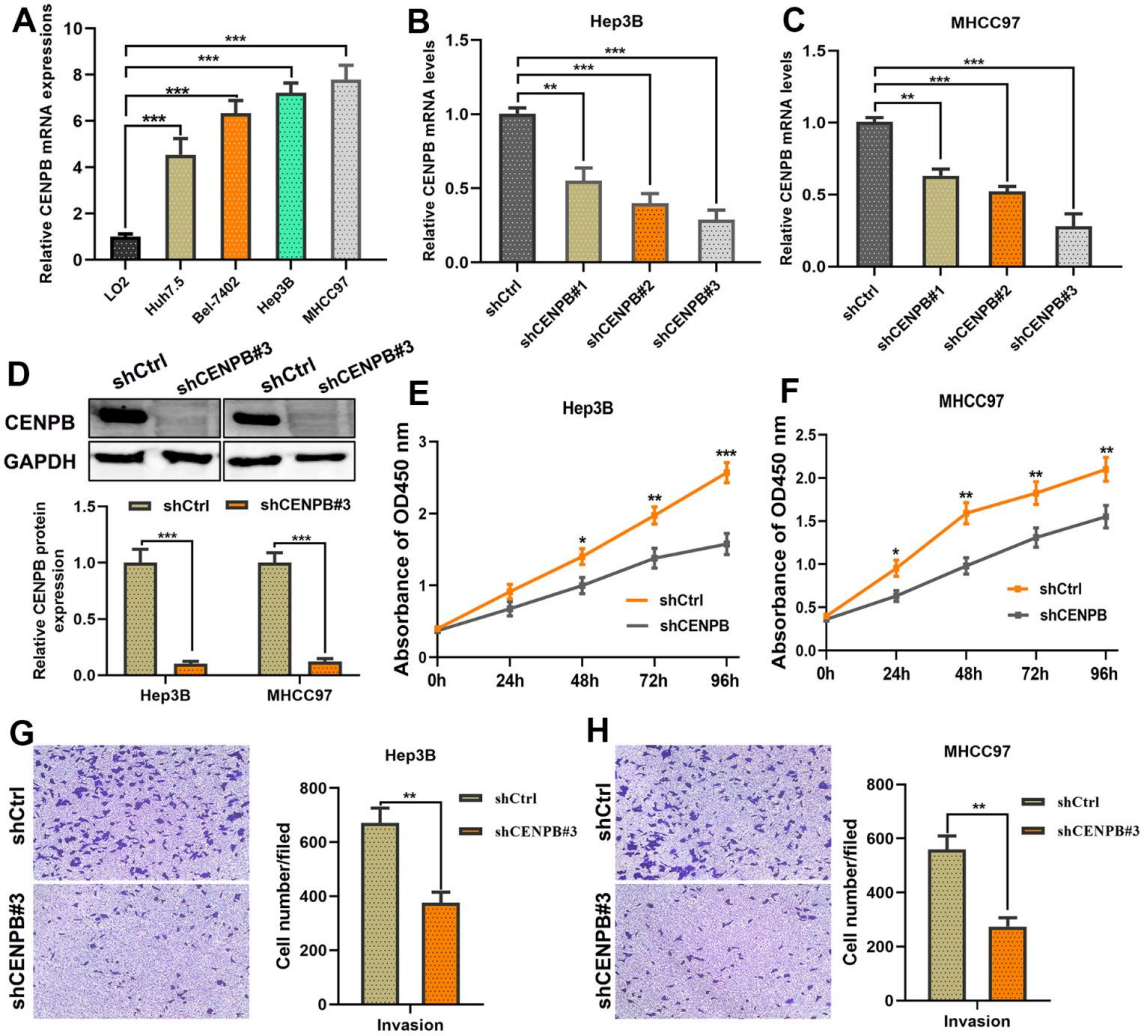
**CENPB knockdown inhibited the proliferation and migration capacities of HCC cells.** (**A**) Expression of CENPB was upregulated in HCC cell lines compared to normal liver cells. (**B**, **C**) Transfection of shCENPB resulted in a decrease in CENPB mRNA levels in Hep3B (**B**) and MHCC97 (**C**) cell lines. (**D**) Western blot assay confirmed the inhibitory effects of shCENPB#3 on CENPB protein expression in Hep3B and MHCC97 cells. (**E**, **F**) CCK-8 assays demonstrated that knockdown of CENPB suppressed the viability of Hep3B (**E**) and MHCC97 (**F**) cells. (**G**, **H**) Transwell assays showed representative images and quantified analysis of cell invasion in Hep3B (**G**) and MHCC97 (**H**) cells transfected with shCtrl or shCENPB#3. *P < 0.05, **P < 0.01, ***P < 0.001. ns: not statistically significant.

Next, we conducted *in vitro* experiments to investigate the impact of CENPB expression on cell proliferative and invasive capabilities. qPCR results revealed significantly higher expression of CENPB in four HCC cell lines compared to LO2 (Figure 5A). Among four HCC cell lines, Hep3B and MHCC97 cell lines exhibited higher mRNA levels, and thus were selected for further experiments. Subsequently, we achieved downregulation of CENPB expression in Hep3B and MHCC97 cell lines by transfecting three different siRNAs, and the knockdown efficiency was investigated by qPCR and Western blot assays. qPCR assay revealed that shCENPB#3 exhibited the best downregulation efficient in both cell lines and was therefore chosen for subsequent cellular experiments (Figure 5B, 5C). Western blot assay validated the excellent knockdown efficiency of shCENPB#3 (Figure 5D). The results of the CCK-8 assay showed a significant inhibition of cell proliferation in HCC cell lines upon downregulation of CENPB expression (Figure 5E, 5F). Likewise, the Transwell assay demonstrated a remarkable suppression of invasion capacity in both Hep3B and MHCC97 cell lines with decreased CENPB expression by transfecting shCENPB#3 (Figure 5G, 5H).

### miR-29a may act as a suppressor for HCC by negatively regulating CENPB expression

**Figure 6:**
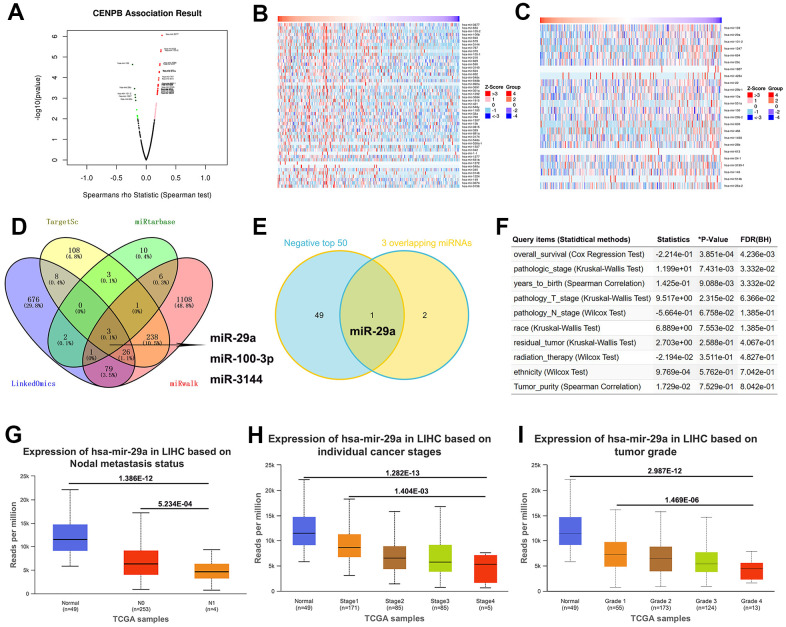
**miR-29a may act as a suppressor for HCC by negatively regulating CENPB expression.** (**A**) The volcano plot revealed positively correlated and negatively correlated miRNAs with CENPB expression in the LinkedOmics database. (**B**, **C**) Heatmaps illustrating the top 50 miRNAs showing positive (**B**) and negative (**C**) correlations with CENPB expression. (**D**) Three overlapping miRNAs: miR-29a, miR-100-3p, and miR-3144 were identified from the LinkedOmics, TargetScan, miRtarbase, and miRwalk databases. (**E**) The Venny diagram exhibited that miR-29a is overlapping in “3 Common miRNAs” and “CENPB Negatively Correlated Significant miRNAs (top 50)”. (**F**) Association of miR-29a expression with clinicopathologic outcomes in HCC patients in the LinkedOmics database. (**G**) miR-29a expression is decreased in in HCC tissue, with a further decrease observed in metastatic tissues. (**H**, **I**) A gradual decrease in miR-29a expression as the tumor stage (**H**) and pathological grade (**I**) increased.

Presently, a considerable body of research indicates that miRNAs exert a crucial role in the diagnosis and treatment of cancer by suppressing the expression of key relevant genes [15, 16]. We utilized bioinformatics analyses to identify miRNAs that modulate the expression of CENPB. The volcano plot revealed 504 miRNAs positively correlated and 289 miRNAs negatively correlated with CENPB expression in the LinkedOmics database (Figure 6A). Moreover, we presented a heatmap illustrating the top 50 miRNAs showing positive and negative correlations with CENPB expression (Figure 6B, 6C). In addition, we conducted correlation analyses in three other databases to identify miRNAs that regulate CENPB expression. Subsequently, by taking the intersection of the results from these four databases, we identified three miRNAs: miR-29a, miR-100-3p, and miR-3144 (Figure 6D). To further narrow down our focus, we intersected these three miRNAs with the top 50 negatively correlated miRNAs identified in the LinkedOmics database, resulting in the selection of miR-29a as the target miRNA (Figure 6E). The correlation analysis conducted in the LinkedOmics database indicated a significant correlation between the expression of miR-29a and Overall_survival (*P*=3.851E-04), pathologic_stage (*P*=7.431E-03), years_to_birth (*P*=9.088E-03), and pathologic_T_stage (*P*=2.315E-02) in patients with HCC (Figure 6F). Additionally, the findings from the UALCAN database revealed a decrease in miR-29a expression in HCC tissue, with a further decrease observed in metastatic tissue (Figure 6G). Interestingly, there was a gradual decrease in miR-29a expression as the tumor stage and pathological grade increased. These results suggest that the reduced expression of miR-29a in HCC may be associated with an unfavorable prognosis (Figure 6H, 6I).

### CENPB is directly negatively regulated by miR-29a in HCC cells

**Figure 7:**
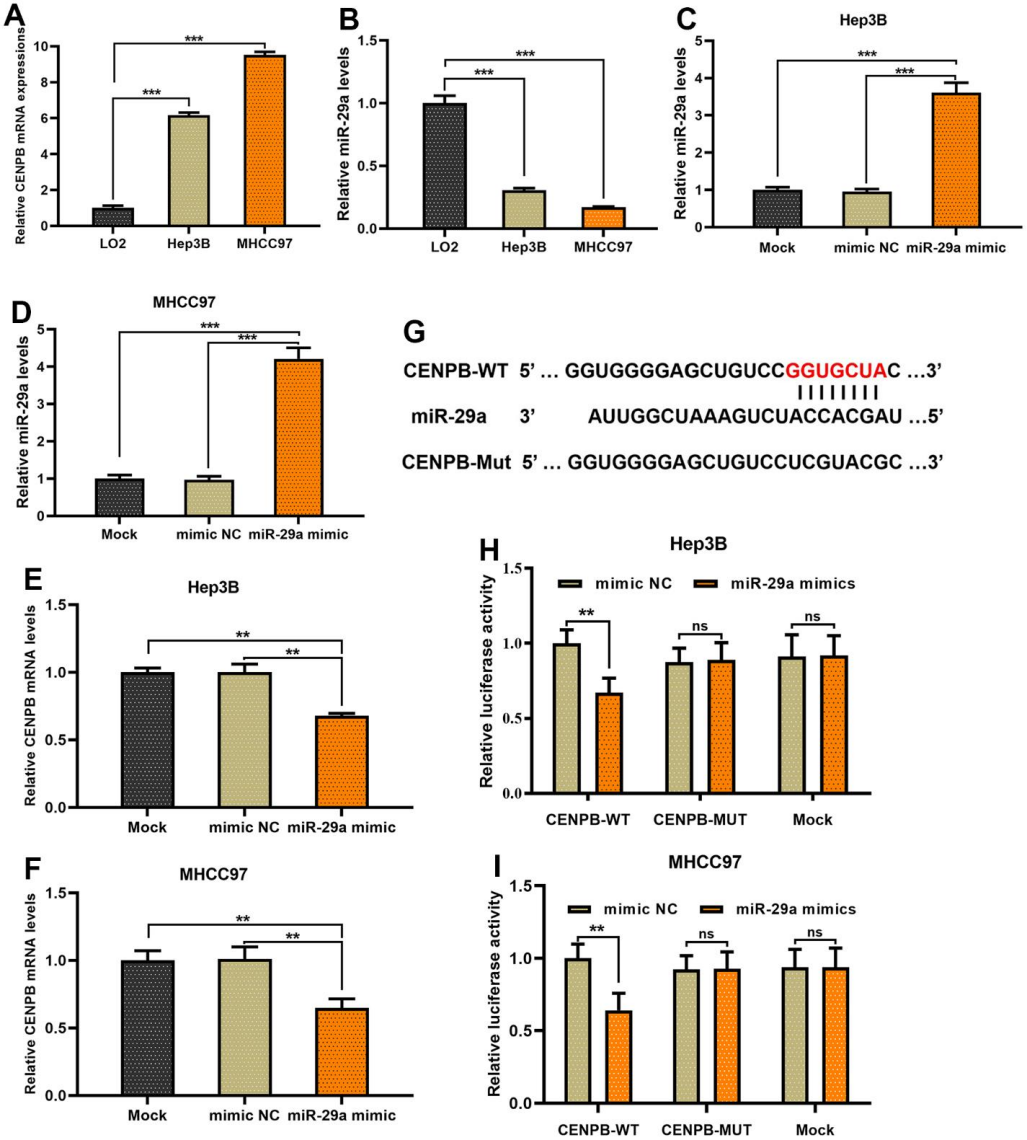
**CENPB is directly negatively regulated by miR-29a in HCC cells.** (**A**, **B**) The expression of CENPB increased gradually in LO2, Hep3B, and MHCC97 cell lines (**A**), whereas miR-29a expression decreased (**B**). (**C**, **D**) miR-29a mimics were transfected into Hep3B (**C**) and MHCC97 (**D**) cell lines, and the successful transfection was confirmed through qPCR assays. (**E**, **F**) The transfection of miR-29a mimics resulted in a reduction of CENPB expression in both Hep3B (**E**) and MHCC97 (**F**) cell lines. (**G**) The 3’UTR of CENPB was identified as the binding site for the 5’UTR of miR-29a. (**H**, **I**) A dual-luciferase gene reporter assay revealed the transfection of miR-29a mimics led to a decrease in luciferase activity in CENPB-WT Hep3B (**H**) and MHCC97 (**I**) cells.

Subsequently, we proceeded with *in vitro* experiments to examine the regulatory role of miR-29a on CENPB. The expression of CENPB increased gradually in LO2, Hep3B, and MHCC97 cell lines, whereas miR-29a expression decreased (Figure 7A, 7B). To enhance the expression of miR-29a, we transfected miR-29a mimics into Hep3B and MHCC97 cell lines, and the successful transfection was confirmed through qPCR assays (Figure 7C, 7D). Remarkably, the transfection of miR-29a mimics resulted in a reduction of CENPB expression in both Hep3B and MHCC97 cell lines (Figure 7E, 7F). These findings verified the inverse association between miR-29a and CENPB expression in HCC. As depicted in Figure 7G, the 3’UTR of CENPB was identified as the binding site for the 5’UTR of miR-29a (Figure 7G). Thereafter, we performed a dual-luciferase gene reporter assay to further validate the direct negative regulatory effect of miR-29a on CENPB. The outcomes revealed that the transfection of miR-29a mimics led to a decrease in luciferase activity in CENPB-WT cells, while no significant alteration was observed in luciferase activity in CENPB-MUT and Mock cells (Figure 7H, 7I). Additionally, consistent outcomes were obtained in Hep3B and MHCC97 cell lines, indicating the direct and negative regulatory role of miR-29a in CENPB expression.

## MATERIALS AND METHODS

### Data acquisition and processing in public databases

We downloaded gene expression data from the TCGA database and corresponding clinical information of HCC patients through the UCSC Xena website [17]. Additionally, two HCC datasets (GSE54236 and GSE76427) were downloaded from the GEO database. We investigated the expression of CENPB in HCC and adjacent normal liver tissues separately in the three datasets. The GSE data set must contain complete gene expression data of HCC patients as well as complete clinical data and prognostic information. In addition, the number of patients in each data set should be greater than 50. Receiver operating characteristic (ROC) curves and the corresponding area under the curve (AUC) were used to evaluate the clinical diagnostic value of CENPB. Next, we conducted survival analysis in the GEPIA and Kaplan-Meier plotter databases to investigate the correlation between CENPB expression and OS and RFS.

### Patients and collection of pathological specimens

We collected paraffin-embedded tissue specimens from 490 patients who underwent curative liver resection for hepatocellular carcinoma at Jiangxi Provincial People’s Hospital between January 2013 and December 2018. Corresponding clinical and pathological data, as well as prognosis information, were also collected. All patients included in the study had to meet the following criteria: 1. Lesion confined to one liver lobe; 2. Patients underwent open liver resection surgery; 3. Postoperative pathological examination confirmed hepatocellular carcinoma; 4. No extrahepatic metastasis; 5. Metastatic liver cancer; 6. No prior or postoperative tumor-related treatment was received. Immunohistochemical staining was used to assess the expression of CENPB in HCC tissue. Exclusion criteria included: 1. Patients who died within one week after surgery due to non-surgical factors; 2. Preoperative Child-Pugh class C; 3. Postoperative pathological confirmation of extrahepatic metastasis or mixed-type liver cancer; 4. Postoperative receipt of other types of anti-tumor treatment, such as immunotherapy, targeted therapy, etc.

### Immunohistochemistry

Immunohistochemical (IHC) staining was employed to evaluate the expression of CENPB protein in HCC tissue. Paraffin-embedded HCC tissue from 490 patients was cut into 4-micron thick tissue sections, followed by immunohistochemical staining. The detailed protocol for immunohistochemical staining could be found in a previously published article. The immunohistochemical staining results were independently evaluated by two experienced pathologists who were blinded to the diagnosis and clinical information of the patients. The specific monoclonal antibody used was as follows: Anti-CENPB (ab284394, 1:300, Abcam, UK). The immunohistochemical staining results were interpreted based on the following scoring system: 0 score, no positive stained cells; 1 score, less than 25% of cells stained positive; 2 score, less than 50% of cells stained positive; 3 score, less than 75% of cells stained positive; 4 score, more than 75% of cells stained positive. ImageJ software was used to calculate the IHC optical density score, thereby quantifying the protein expression of CENPB.

### Prognostic value of CENPB protein expression and nomogram construction

According to the immunohistochemistry staining, 490 patients with HCC were divided into high-expression and low-expression groups based on CENPB expression levels. Patients with a score of 0, 1, or 2 were classified into the low expression group, while patients with a score of 3 or 4 were classified into the high expression group. Univariate and multivariate Cox regression analyses were conducted to identify independent risk factors for overall survival (OS) and recurrence-free survival (RFS). All independent predictive factors were integrated into a nomogram for quantitative prediction of 1-year, 3-year, and 5-year OS and RFS. Subsequently, calibration curves and time-dependent ROC curves were used to evaluate the predictive ability of the nomogram at different time points.

### Identification of miRNAs associated with CENPB expression

We conducted a comprehensive analysis of miRNAs that were associated with the expression of CENPB using four databases, namely LinkedOmics, TargetScan, miRtarbase, and miRwalk. By comparing the miRNA data from these databases, we identified a set of miRNAs that were implicated in the regulation of CENPB expression. Furthermore, we narrowed down our focus by considering the intersection between these miRNAs and the top 50 miRNAs negatively correlated with CENPB expression in the LinkedOmics database, as they are particularly influential in regulating CENPB expression. Subsequently, we explored the clinical prognostic value of these identified miRNAs in HCC using the LinkedOmics database. Additionally, we utilized the UALCAN database to investigate the expression profiles of these miRNAs in normal liver tissue and different pathological stages of HCC.

### Cell culture and plasmid transfection

Human hepatocyte cell line LO2 and hepatoma cell lines Huh7.5, Bel-7402, Hep3B, and MHCC97 were purchased from the National Certification Cell Bank (Shanghai, China). All cells were cultured in DMEM medium supplemented with 10% fetal bovine serum (FBS) and 1% antibiotics (100 U/mL penicillin, 100 μg/mL streptomycin). The cell culture process was conducted in a constant temperature incubator at 37° C with 5% CO2. The lentivirus carrying three shRNA-CENPB (shCENPB) or shRNA-CENPB negative control sequences (shCtrl) were transfected into Hep3B and MHCC97 cell lines using Lipofectamine® 3000 transfection reagent. The plasmid transfection process was performed following the guidelines provided by the manufacturer. Three oligonucleotide sequences were design to generate deletion in CENPB: shCENPB#1: 5′- CACCGGATGTTGCAGAACTCCGCCG -3’, shCENPB#2: 5′-CACCGCGTACTTGCGCTCCGACGCC -3’; and shCENPB#3: 5′-CACCGGGAAGTAGCAGAGCGAGGGC -3’.

### RNA extraction and qRT-PCR

After 48 h of transfection, cells were harvested. Total RNA extraction was performed using RNAiso Plus (TaKaRa, 9109, China) following the manufacturer’s instructions. The extracted total RNA was dissolved in RNase-free water. Subsequently, cDNA synthesis was carried out using gDNA Purge (Novoprotein, E047-01A, China) and stored at -20° C. qPCR reactions were performed on the ABI 7500 Fast Real-Time PCR System (Applied Biosystems, USA) using NovoStart® SYBR qPCR SuperMix Plus (Novoprotein, E096-01B, China), and the increase in fluorescence signals was recorded. GAPDH and U6 were used as reference genes for CENPB and miR-29a, respectively. The 2^-ΔΔCt^ method was employed to analyze the relative expression levels of CENPB and miR-29a. The primer sequences are provided in Table 5.

**Table 5:** Primers sequences for qRT-PCR.

**Name**	**Sequences**
miR-29a	Forward: 5’- CTGGTGTCGTGGAATTCAGTTGA-3’
	Reverse: 5’-CCTGGCTCCTCACTTGGC-3’
CENPB	Forward: 5’- ATTCAGACAGTGAGGAAGAGGACG-3’
	Reverse: 5’- CATCAATGGGGAAGGAGGTCAG-3’
U6	Forward: 5’- GGAACGATACAGAGAAGATTAGCA-3’
	Reverse: 5’- GTGCAGGGTCCGACCT-3’
GAPDH	Forward: 5’- AAGGTGAAGGTCGGAGTCAAC-3’
	Reverse: 5’- GTTGAGGTCAATGAAGGGGTC-3’

### Cell counting kit-8 assay

We conducted CCK-8 assays to determine the effect of CENPB on the proliferation capacity of Hep3B and MHCC97 cells. After transfection for 48 h, the cells were harvested and seeded in a 96-well plate at a concentration of 2×10^3^ cells per well. Subsequently, the plate was incubated at 37° C with 5% CO2 for 24 h, 48 h, 72 h, and 96 h, respectively. After the completion of the incubation period, the culture medium was discarded, and the CCK-8 reagent was added. The absorbance of the cells was measured at 450 nm using an enzyme-linked immunosorbent assay (ELISA) reader to evaluate their proliferation capacity.

### Transwell invasion assay

We conducted the Transwell assay to evaluate the effect of CENPB on the invasive capability of Hep3B and MHCC97 cells. After 48 h of transfection, the cells were harvested and cultured in DMEM medium containing 10% FBS until reaching the desired cell density. The upper chamber of the Transwell (8 μm pore size) was coated with a layer of Matrigel (1 mg/mL, 100 μl). The cells were suspended at a concentration of 5 × 10^3^ cells/well in serum-free medium and added to the upper chamber, ensuring even distribution of cells. The Transwell chambers were then incubated at 37° C with 5% CO2 for 24 h. Subsequently, the non-invasive cells in the upper chamber were gently removed using a cotton swab to eliminate cells that did not pass through the Transwell pores. The cells in the lower chamber were fixed with 4% paraformaldehyde for 15 minutes and then stained with toluidine blue for 15 minutes. Finally, under a microscope, the invasive cells in the lower chamber were counted, and the cell numbers were recorded.

### Western blot

We conducted Western blot experiments to detect the protein expression of CENPB in Hep3B and MHCC97 cells. After 48 h of transfection, the cells were harvested and cultured in DMEM medium containing 10% FBS until reaching the desired cell density. RIPA buffer was used to lyse the cells, and the cell lysates were centrifuged to collect the total cellular protein supernatant. The cell lysates were mixed with protein loading buffer and heated to 95° C, then loaded onto a protein gel for electrophoresis separation. The separated proteins were then transferred onto a PVDF membrane using the electrophoresis transfer method. The PVDF membrane was subsequently incubated with the primary antibody (ab284394, 1:2000, Abcam, UK) overnight. After incubation with the secondary antibody (1:5000, Solarbio, China), the protein bands on the PVDF membrane were visualized using a chemiluminescence imaging system (Bio-Rad, USA) and quantified using image analysis software (Bio-Rad, USA).

### Dual-luciferase reporter assay

We performed a dual-luciferase reporter assay to investigate the reciprocal regulation between miR-29a and CENPB. The miR-29a mimic sequence was cloned into the upstream region of the firefly luciferase reporter vector to generate the miR-29a reporter vector. The wild-type (WT) and mutant (MUT) CENPB 3’-UTR sequences were cloned into the psicheck2.0 vector (GenePharma, Shanghai, China). The miR-29a mimic and CENPB expression vector were co-transfected with the psicheck2.0 vector containing the WT or MUT CENPB 3’-UTR into Hep3B and MHCC97 cells using Lipofectamine 3000 (Invitrogen, USA). Cells transfected with empty vector were defined as the mock group. After 48 h of transfection, the luciferase activity in transfected cells was measured using the Duo-LiteTM Luciferase Assay System (Vazyme Biotech Co., Ltd, China) according to the manufacturer’s instructions.

### Statistical analysis

Statistical analysis was performed using SPSS 19.0 software. Graphical representation of the data was generated using GraphPad 8.0 software. All experiments were conducted independently for a minimum of three times. Student’s t-test and Mann-Whitney U test were used to analyze the comparisons between two groups. Clinical and pathological characteristic data were analyzed using a two-tailed chi-square test. Univariate and multivariate Cox regression analysis models were used to identify independent prognostic factors. A significance level of P < 0.05 was considered statistically significant, indicating a significant difference.

### Availability of data and materials

The datasets generated and/or analyzed during the current study are available from the corresponding author on reasonable request.

## DISCUSSION

Malignant neoplasms pose a substantial public health challenge for medical researchers worldwide [18]. HCC is a prevalent malignant solid tumor in the human gastrointestinal system [19]. Epidemiological data indicate a rapid rise in HCC incidence, elevating it to a global public health concern [20]. Despite notable advancements in treatment modalities like surgery, chemotherapy, and radiotherapy, the overall prognosis for HCC remains unsatisfactory [21]. Extensive research has shed light on the intricate mechanisms driving cancer development, with HCC progression resulting from the interplay of multiple factors, including oncogene overexpression, dysregulated proliferation control, immune evasion, apoptosis inhibition, and protein interactions [22–25]. These factors collectively contribute to the onset and metastasis of cancer. The development of HCC involves the modulation of numerous genes, many of which hold clinical significance in tumors and serve as valuable biomarkers for cancer diagnosis and prognosis [26, 27]. Moreover, these genes exhibit distinct biological functions during tumor progression, influencing the proliferation processes [28]. Consequently, they have emerged as therapeutic targets for tumor-specific interventions aimed at modulating these functional molecules [29, 30].

CENPB is a protein that plays a crucial role in the centromeric region of chromosomes and is closely involved in the proper separation of chromosomes during cell division [31]. CENPB primarily binds to the centromeric α-satellite DNA sequences and participates in the assembly of kinetochores, which are protein complexes that connect chromosomes to the mitotic spindle during cell division [32]. CENPB is also involved in regulating some cellular processes such as gene expression, DNA replication, and DNA repair [33]. Additionally, emerging research suggests that CENPB may be involved in the development of cancer. In various types of cancer, CENPB expression is abnormal or dysregulated, and it is associated with tumor progression and poor prognosis [34–36]. CENPB may promote tumor development by affecting chromosomal stability and regulating gene expression. A large-scale parallel sequencing study has found that CENPB protein assists in the formation of kinetochores during mitosis and is involved in the WNT signaling pathway, thereby promoting the progression of colon cancer [10].

Our research revealed that CENPB mRNA and protein levels are significantly upregulated in HCC tissues. Notably, as the tumor stage and pathological grade advance, there is a gradual increase in CENPB mRNA and protein expression, indicating a strong association between CENPB expression and tumor prognosis. In addition, elevated CENPB expression has been shown to be independent risk factors for poorer OS and RFS. Utilizing these findings, we developed precise nomograms to predict OS and RFS at 1-year, 3-year, and 5-year intervals. The accuracy of our predictions was validated through time-dependent ROC analysis and calibration curves. Although these nomograms demonstrated accurate predictive capabilities and were based on a relatively large sample size of 490 HCC patients, further confirmation of their clinical applicability would necessitate multi-center studies with larger sample sizes, considering that the current results were obtained from a single-center dataset.

The imbalance in the expression of miRNAs has been proven to have a significant impact on diverse signaling pathways and mechanisms underlying many cancers [37, 38]. More than half of the miRNAs found in the human genome are situated in genomic regions associated with cancer, and they contribute to the development of tumors either as tumor suppressors or oncogenes [39]. In our research, we employed bioinformatics analysis to identify overlapping miRNAs that regulate CENPB expression across multiple databases. Among them, miR-29a emerged as the most highly correlated one. Furthermore, our investigations spanning multiple databases demonstrated a negative association between miR-29a and CENPB expression in HCC. Interestingly, we observed that decreased expression of miR-29a in HCC tissues correlated with unfavorable clinical pathological outcomes, suggesting its pro-oncogenic role. To validate this, we conducted dual-luciferase reporter gene assays, confirming the direct negative regulatory effect of miR-29a on CENPB.

Aberrant expression of miR-29a has been identified in various types of cancer [40]. It may exhibit a double-edged sword effect in different tumor types [41]. In bladder, prostate, and various gastrointestinal tumor tissues, miR-29a is found to be significantly downregulated, and its low expression plays a pro-oncogenic role [42–44]. However, in cholangiocarcinoma and breast cancer, miR-29a demonstrates upregulation, promoting tumor development [45, 46]. In HCC, Liu et al. found that miR-29a expression is reduced and promotes tumor proliferation by negatively regulating the expression of IFITM3 [47]. Cui et al. demonstrated that miR-29a is involved in the progression of non-alcoholic fatty liver disease to HCC by regulating the NOTCH2 axis [48]. Our research aligns with their findings as we also observed low expression of miR-29a in HCC tissues. However, we identified an additional downstream target gene, CENPB, which further enhances our understanding of the molecular mechanisms by which miR-29a is involved in HCC progression. Hence, the simultaneous targeting of the miR-29a/CENPB axis holds promise as a novel therapeutic strategy for hepatocellular carcinoma. Nevertheless, additional experimental evidence is required to substantiate this concept.

## CONCLUSIONS

In summary, we found that CENPB mRNA and protein were highly expressed in HCC tissues and were independent risk factors for poor OS and RFS. *In vitro* experiments revealed that downregulation of CENPB expression could potentially inhibit the proliferation and invasive capabilities of HCC cells. Subsequently, we constructed quantitative prediction nomograms for patient OS and RFS and validated their effectiveness. Through bioinformatics analysis, we identified miR-29a as a potential upstream gene regulating CENPB expression, with its decreased expression indicating poorer prognosis for patients. Dual-luciferase reporter assays confirmed the direct negative regulatory effect of miR-29a on CENPB. Therefore, targeting the miR-29a/CENPB axis in combination may provide a new approach for the treatment of HCC.
